# Potential Risk of Higenamine Misuse in Sports: Evaluation of Lotus Plumule Extract Products and a Human Study

**DOI:** 10.3390/nu12020285

**Published:** 2020-01-21

**Authors:** Ching-Chi Yen, Chun-Wei Tung, Chih-Wei Chang, Chin-Chuan Tsai, Mei-Chich Hsu, Yu-Tse Wu

**Affiliations:** 1 School of Pharmacy, Kaohsiung Medical University, Kaohsiung 807, Taiwan; date0315@hotmail.com (C.-C.Y.); wxes9050304@gmail.com (C.-W.C.); 2 Graduate Institute of Data Science, College of Management, Taipei Medical University, Taipei 106, Taiwan; cwtung@tmu.edu.tw; 3 National Institute of Environmental Health Sciences, National Health Research Institutes, Miaoli County, 350, Taiwan; 4 School of Chinese Medicine for Post-Baccalaureate, I-Shou University, Kaohsiung 840, Taiwan; tsaicc@isu.edu.tw; 5 Chinese Medicine Department, E-Da Hospital, Kaohsiung 824, Taiwan; 6 Department of Sports Medicine, Kaohsiung Medical University, Kaohsiung 807, Taiwan; 7 Department of Medical Research, Kaohsiung Medical University Hospital, Kaohsiung 807, Taiwan; 8 Substance and Behavior Addiction Research Center, Kaohsiung Medical University, Kaohsiung 807, Taiwan; 9 Drug Development and Value Creation Research Center, Kaohsiung Medical University, Kaohsiung 807, Taiwan

**Keywords:** higenamine, lotus plumule, microwave-assisted extraction, doping

## Abstract

Since 2017, higenamine has been added to the World Anti-Doping Agency (WADA) prohibited list as a β_2_-agonist prohibited at all times for sportspersons. According to WADA’s report, positive cases of higenamine misuse have been increasing yearly. However, higenamine occurs naturally in the Chinese herb lotus plumule—the green embryo of lotus (*Nelumbo nucifera* Gaertn) seeds—commercially available as concentrated powder on the Asian market. This study evaluated the major phytochemical components of lotus plumule products using an appropriate extraction method, followed by a human study in which the products were orally administered in multiple doses to investigate the risk of doping violations. Comparing various extraction methods revealed that optimized microwave-assisted extraction exhibited the highest extraction efficiency (extraction time, 26 min; power, 1046 W; and temperature, 120 °C). Subsequently, the alkaloids in lotus plumule products were quantitatively confirmed and compared. Human study participants (*n* = 6) consumed 0.8 g of lotus plumule (equivalent to 679.6 μg of higenamine) three times daily for three consecutive days. All participants’ urinary higenamine concentrations exceeded the WADA reporting cut-off of 10.0 ng/mL. Accordingly, lotus plumule consumption may engender adverse analytical findings regarding higenamine. Athletes should avoid consuming lotus plumule-containing products during in- and out-of-competition periods.

## 1. Introduction

In competitive sports, doping refers to the use of banned athletic performance—enhancing drugs/materials by athletic competitors. The use of doping agents is generally considered both unhealthy and contrary to the ethics of sport. Accordingly, the World Anti-Doping Agency (WADA) has been established with the mission of leading a collaborative worldwide movement for doping-free sport, and its activities focus on the responsibilities provided by the World Anti-Doping Code. One such responsibility is to publish a prohibited list, which identifies the substances and methods prohibited in- and out-of-competition, particularly in sports [1]. Since 2004, WADA has annually updated the prohibited list. If a substance or method is deemed to meet two of the following three criteria, it can be added to the list: (1) It has the potential to enhance or enhances sport performance, (2) it represents an actual or potential health risk to the athlete, and (3) it violates the spirit of sport described in the introduction to the code. The presence of a prohibited substance or its metabolites or markers (including elevated quantities of endogenous substances) in a specimen or evidence of the use of a prohibited method will be considered an adverse analytical finding (AAF) [2].

Dietary supplements are used by athletes in every aspect of the sport, reflecting their popularity. Approximately half of adults in the United States regularly consume different types of dietary supplements [3]. Regardless of regional, cultural, or economic differences, a similar prevalence is likely to be observed in many other countries. In sports, products described as “supplements” can target various roles in an athlete’s performance plan [4]. Athletes may consume specific nutrients to maintain optimal health, manage micronutrient deficiencies, and meet energy and macronutrient requirements, which may be difficult to achieve by daily diets alone. Other motivations that supplement consumption as reported by athletes include direct enhancement of performance, manipulation of physique, mitigation of musculoskeletal pain, acceleration of recovery from injury, and improvement of mood. However, some products may contain doping substances, and some herbal extract-based products may be contaminated with agents prohibited in sport. In 1999, Ros et al. reported that the urine of a Dutch professional cyclist who had consumed a herbal food supplement called “Limiet 65 slankheidsdruppels” was discovered to be positive for norpseudoephedrine (20.2 µg/mL) during a doping control [5]. Both ephedrine and its derivatives such as cathine, methylephedrine, and pseudoephedrine are considered doping substances, and relatively high doses of these substances would exert several harmful effects on the body’s health [6]. In addition, consuming *Papaver somniferum* [7] and a Chinese herbal medicine called “LiDa Dai Dai Hua Jiao Nang” [8] could cause athletes to fail a doping test. An AAF resulting from the intake of herbal medicine can be caused by an athlete’s poor knowledge of banned substances indicated on a product label, due to the fact that the labeled ingredients indeed contain banned substances, or due to the athlete’s limited investigation of herbal ingredients.

Lotus plumule, the green embryo of lotus (*Nelumbo nucifera* Gaertn) seeds with a bitter taste, has been widely consumed as a tea by Asian people. As a traditional medicine, lotus plumule is used for treating nervous disorders, insomnia, high fever (with restlessness), and cardiovascular disease [9]. Lotus plumule possesses several pharmacological properties, which are generally considered to be related to its active components, especially flavonoids and alkaloids [10,11]. Moreover, lotus plumule contains higenamine, liensinine, dauricine, isoliensinine, neferine, and nuciferine, which exhibit high bioactivity and favorable health care function [12,13]. In particular, higenamine was added to the WADA prohibited list in 2017 under the S3 category as a nonselective β2-agonist. Higenamine is a natural constituent of several traditional botanical remedies and is listed as an ingredient in over-the-counter weight loss and sports supplements sold in the United States [14]; therefore, different dietary supplements used as fat burners could potentially contain this ingredient. WADA established a criterion for higenamine as a banned substance, according to which its analytical finding should not be reported at levels below 10.0 ng/mL (i.e., 50% of the minimum required performance level for β_2_ agonists) [15]. However, concerns have been raised regarding the potential cause of increased cases of unintentional higenamine doping in the Asian region. Masato et al. [16] investigated higenamine levels in human urine after the administration of a throat lozenge containing Nandina domestica fruit. They observed that urinary concentrations of higenamine after intake did not reach the cut-off level of 10.0 ng/mL. In the Asian market, lotus plumule is usually available as a concentrated powder, especially in Taiwan; therefore, athletes can easily obtain such products. However, the literature contains only preliminary data regarding this topic; moreover, only a few studies have addressed the topic. Accordingly, the present study assessed the potential risk of lotus plumule consumption by athletes.

Based on this background information, the aims of this study were to investigate both the constituents present in lotus plumule and its implications on doping violation. We first quantitatively analyzed the concentrations of higenamine and related alkaloids in selected products of lotus plumule in Taiwan. For this quantitative analysis, we applied the design of experiment (DOE) method to optimize a microwave-assisted extraction (MAE) process by using response surface methodology (RSM). In addition, chemometric tools, namely hierarchical cluster analysis (HCA) and principal component analysis (PCA), were applied to analyze quality variations and multivariate associations in the studied products. Finally, we conducted a human study with a multiple-administration design for three consecutive days to determine whether supplementation with concentrated herbal extract products (HEPs) of lotus plumule could cause a urinary concentration higher than 10.0 ng/mL and result in an AAF as defined by WADA.

## 2. Materials and Methods 

### 2.1. Chemicals and Reagents

Higenamine hydrochloride (6,7-dihydroxy-1-(4-hydroxybenzyl)-1,2,3,4-tetrahydroisoquinoline hydrochlorid; purity ≥ 95%) was obtained from Combi-Blocks Inc. (San Diego, CA, USA). Liensinine, dauricine, isoliensinine, neferine, and nuciferine (purity ≥ 98%) were purchased from Grand Chemical Co. Ltd. (Miaoli, Taiwan). Analytical-grade acetonitrile was supplied by J.T. Baker Avantor Performance Materials, Inc. (Center Valley, PA, USA). Ethanol 95% (v/v) was obtained from Echo Chemical (Miaoli, Taiwan). Sodium phosphate monobasic was supplied by Sigma-Aldrich (St. Louis, MO, USA). All other chemicals used in the study were of analytical grade. Pure water was obtained by using the Milli-Q system (Millipore, Bedford, MA, USA).

### 2.2. Extraction Procedure

#### 2.2.1. MAE Process

MAE was performed using a MARS 5 microwave system (CEM, Matthews, NC, USA). During extraction, time, power, and temperature could be controlled. A preliminary study was performed to determine the effect of the solid-to-solvent ratio on the percent yield of total alkaloids from lotus plumule. A solid-to-solvent ratio of 1:20 provided the maximum concentration of alkaloids (data not shown). Therefore, the extraction was performed by placing 1.0 g of a ground sample of lotus plumule in a vessel with 20 mL of extraction solvent.

#### 2.2.2. DOE Study for MAE

The Box–Behnken design (BBD) was used as the model to determine the polynomial relationship between variables and selected responses (dependent variables, e.g., higenamine concentration). RSM was used to determine the optimal conditions for effectively extracting alkaloids from lotus plumule. The effects of three independent variables, namely extraction time (5–30 min), microwave power (500–1500 W), and temperature (60–120 °C), on the dependent variables were investigated to determine the optimal conditions for maximizing the percent yield of the selected alkaloids from lotus plumule. For each variable, the corresponding low, middle, and high ranges were designated as −1, 0, and +1, respectively (Table 1). After the variables and their ranges were determined, experiments were established based on the BBD. The complete design comprised 17 experiments with five replicates of the central points to fit the full quadratic equation model. For the polynomial equation, the dependent variables were the extraction yields of higenamine, liensinine, daurcine, isoliensinine, and neferine. After the extraction process, the extracts were immediately cooled to room temperature, filtered through filter paper (Advantec, Tokyo, Japan), and quantitatively adjusted to 20 mL with 95% (v/v) ethanol. The samples were filtered through a 0.45 μm syringe filter and diluted up to 20-fold with 50% (v/v) methanol, after which high-performance liquid chromatography (HPLC) analysis was performed.

#### 2.2.3. Comparison with Other Extraction Methods

The MAE method was compared with the following three extraction methods: Soxhlet extraction (SE), heat reflux extraction (HRE), and ultrasound-assisted extraction (UAE). All relevant experiments were performed in triplicate.

SE: The extraction process was performed using a Soxhlet apparatus. Specifically, 10.0 g of a ground sample of lotus plumule was placed in a paper thimble (28 × 100 mm; Advantec). The extraction was performed for 8 h with 200 mL of 95% (v/v) ethanol at 90 °C. After cooling, the extract was filtered through filter paper and quantitatively adjusted to 200 mL with 95% (v/v) ethanol. The subsequent processes were the same as those in the methods described in the preceding section.

HRE: In this extraction process, 5.0 g of a ground sample of lotus plumule was mixed with 100 mL of 95% (v/v) ethanol in a round-bottom flask and boiled at 90 °C for 4 h. After cooling, the extract was filtered through filter paper and quantitatively adjusted to 100 mL with 95% (v/v) ethanol. The subsequent processes were the same as those in the methods described in the preceding sections.

UAE: An ultrasonic bath (Branson 3210) was used to perform UAE. In this process, 1.0 g of a ground sample of lotus plumule was mixed with 20 mL of 95% (v/v) ethanol in a tube and ultrasonicated for 30 min. Subsequently, the extract was filtered through filter paper and quantitatively adjusted to 20 mL with 95% (v/v) ethanol. The subsequent processes were the same as those in the methods described in the preceding sections.

### 2.3. Quantitative Determination of Lotus Plumule Products

#### 2.3.1. Chromatographic Conditions and Validation

A Hitachi Chromaster HPLC system (Tokyo, Japan) equipped with a 5160 pump, 5260 autosampler, and 5430 photodiode array ultraviolet (UV) detector was used to validate the chromatographic conditions. All analytical samples were separated using a Luna phenyl-hexyl column (250 × 4.6 mm, i.d. 5 μm; Phenomenex, Torrance, CA, USA). Gradient elution was performed using acetonitrile as solvent A and 0.01 M Na_2_HPO_4_ (pH 2.5 adjusted with orthophosphoric acid) as solvent B filtered through a membrane filter (0.45 μm, Millipore) and sonicated before use. The gradient program was as follows: 0–10 min, 10% A; 10.1–35 min, 15–25% A; 35–40 min, 25–30% A; 40.1–45 min, 90% A; and 45.1–55 min, 10% A. The flow rate of the mobile phase was 1.0 mL/min, and the sample injection volume was 20 μL.

Stock solutions of higenamine, liensinine, dauricine, isoliensinine, neferine, and nuciferine were prepared in methanol (1.0 mg/mL) and stored at −70 °C; they were warmed to room temperature before use. Calibration standards were serially diluted with 50% (v/v) methanol to form the working standard solutions (0.1–25.0 μg/mL). Quality control (QC) samples were also prepared in the same manner. Using least squares linear regression, we obtained a calibration curve (*y* = a*x* + b) by plotting the peak area (*y*) against the concentration (*x*) of the calibration solution. Limit of quantification (LOQ) and limit of detection (LOD) were defined as peaks’ height yielding signal-to-noise (S/N) ratios of 10 and 3, respectively. Moreover, intra- and inter-day precision and accuracy were evaluated through an analysis of variance (ANOVA) based on replicate analysis of QC samples; a standard calibration curve was used for each analytical run. Precision is presented as the relative standard deviation (RSD%) at each concentration level, and accuracy is presented as relative error (RE%).

#### 2.3.2. Preparation of Lotus Plumule Products

Eleven commercially available products in Taiwan containing herbal extract product (HEP) and crude lotus plumule (CLP) were purchased and stored at room temperature until use. All purchased products were well within their expiration dates as provided by the manufacturer. Samples were prepared using the optimized MAE condition and analyzed through the developed HPLC analytical method. Each sample (1.0 g) was mixed with 20 mL of 95% (v/v) ethanol in a vessel and irradiated with microwave power (1046 W) at 120 °C for 26 min. After cooling, the extract was filtered through filter paper and quantitatively adjusted to 20 mL with 95% (v/v) ethanol. The subsequent processes before HPLC analysis were the same as those in the methods described in the preceding sections. All commercial products were subjected to HCA and PCA using R language version 3.1.2 as described previously [17]. Cluster analysis is a multivariate technique that arranges components on the basis of their characteristics. It classifies components on the basis of their similarity in space. As a result, cluster exhibits high homogeneity in the intergroup and high heterogeneity among different groups [18,19]. Regarding HCA, the Euclidean distance was used to calculate the similarity between the vectors of the log-transformed concentrations of the alkaloids that were extracted from all tested products. Subsequently, using the complete-linkage method, we created a hierarchical clustering tree by iteratively merging the most similar clusters. PCA is used to reduce the dimensionality of a dataset consisting of a large number of interrelated variables, while retaining the variation present in the data set as much as possible. This is achieved by converting to a new set of variables which are irrelevant and ordered, so the first few components retain most of the variation in all the original variables [19,20]. Regarding PCA, the vectors of the log-transformed concentrations of all tested products were converted into a set of linearly uncorrelated principal components. The top two principal components with the highest explained variance percentage were then used for visualization and analysis.

### 2.4. Human Study 

#### 2.4.1. Enrollment Criteria and Dosage Regimen

The study protocol was approved by the Institutional Review Board of E-Da Hospital (No. EMRP63107N). A human study was conducted over a period of three days to examine the concentration of higenamine in urine after product administration; the product was administered to six healthy men aged between 25 and 35 years. HEP-3 was selected as the study model because it was determined to have the highest higenamine concentration. Participants who consumed any supplements or medications regularly, had a body mass index that was outside the range of 18.5–24 kg/m^2^, smoked, or consumed alcohol were excluded from the study. Before the study, all participants provided written informed consent. The participants were requested to consume 0.8 g powder of HEP-3 (taken with warm drinking water) three times a day at 8:00, 13:00, and 18:00 for three days consecutively (i.e., days 1, 2, and 3). On the first day, urine specimens were collected before the administration, 1, 2, and 3 h after each administration of the product (i.e., 9:00, 10:00, 11:00, 14:00, 15:00, 16:00, 19:00, 20:00, and 21:00). On the second and third days, urine specimens were collected 1, 2, and 3 h after the last administration of the product (i.e., 19:00, 20:00, and 21:00). The collected urine specimens were stored at −20 °C before analysis.

#### 2.4.2. Urine Sample Preparation and Analysis

Urine samples were collected and underwent enzymatic hydrolysis to evaluate the total amount of higenamine [16]. An aliquot (1 mL) of each urine sample was pipetted into a glass tube, followed by the addition of 12.5 μL of internal standard (IS, dobutamine-*d4*, 500 ng/mL). Moreover, an aliquot (1 mL) of 0.1 M phosphate buffer (pH = 6.0) and 25 μL of *β*-glucuronidase (from *Escherichia coli*) were added and mixed thoroughly under heating at 50 °C for 60 min for hydrolysis. Subsequently, the hydrolyzed sample was applied onto a solid-phase extraction cartridge (DAU, 3 mL, Bond Elut, Agilent). The solid-phase cartridge was washed with 2 mL of deionized water, 2 mL of 0.1 M HCOOH, and 2 mL of methanol. The eluate was collected from 2 mL of a mixture of dichloromethane, isopropyl alcohol, and NH_4_OH (80:20:2, v/v/v). The supernatant organic phase was obtained and dried under nitrogen at 60 °C. The residue was reconstituted using 200 μL of a mixture of 0.02% (v/v) formic acid and methanol (85:15, v/v), and 20 μL was injected into an HPLC tandem mass spectrometry (HPLC-MS/MS) system.

The urine samples were analyzed on an Agilent 1260 series HPLC system (Agilent Technologies, Palo Alto, CA, USA) and an Agilent 6470 triple-quadruple mass spectrometer (Agilent Technologies, Santa Clara, CA, USA) operated in positive ion mode. The analytes were separated using a Supelco Ascentis HP-C18 column (100 × 2.1 mm, id. 3μm; Sigma–Aldrich) and a Supelco Discovery HS-C18 guard column (20 × 2.1 mm; id, 3μm; Sigma–Aldrich). Gradient elution was employed using 0.1% (v/v) formic acid as solvent A and acetonitrile as solvent B. The gradient program was as follows: 0–0.5 min, 95% A; 0.5–6.0 min, 95–5% A; 6.0–6.1 min, 5–95% A; and 6.1–15.0 min, 95% A. The flow rate was set at 0.3 mL/min, and the injection volume was 20 μL. The total run time was 15 min for each sample. In multiple reaction monitoring, the detector response is due to a specific transition of molecular ions to fragments (molecular ions ⟶ fragment). The fragmentations of 272.3 ⟶ 107.0 m/z (CE: 24 eV) and 272.3 ⟶ 161.0 m/z (CE: 16 eV) ion were selected for higenamine, and that of the IS were 306.3 ⟶141.0 m/z (CE: 24 eV) and 306.3 ⟶ 107.0 (CE: 36 eV). The linearity, ranging from 0.5 to 30.0 ng/mL, was validated for higenamine, and the coefficient of determination (*R^2^*) for calibration was determined to be 0.9989. The observed intra- and inter-day accuracy ranged from 89.3% to 96.2%, and the observed precision ranged from 0.6% to 6.3%. The mean percent recovery of higenamine varied from 73.8% to 82.7%. Analysis data are presented in Table S1.

### 2.5. Statistical Analysis

The obtained data are expressed as mean ± standard deviation. ANOVA for RSM was performed using Design-Expert 6.0.3 software (StatEase Inc., Minneapolis, MN, USA). The effects of the different extraction methods on alkaloid yield were compared statistically using ANOVA followed by a post hoc Scheffe test using SPSS v14.0 (SPSS Inc., Chicago, IL, USA). A *p*-value of < 0.05 was considered to indicate a significant difference.

## 3. Results and Discussion

### 3.1. Statistical Analysis and Model Fitting for Lotus Plumule Extraction Using MAE

To optimize the extraction conditions of the target compounds, we adopted a 17-run BBD with three variables (extraction time, microwave power, and temperature) and three levels. Other parameters, namely the amount of dried sample (1.0 g) and solvent volume (20 mL), involved in the extraction remained constant. The actual and coded levels of the independent variables used in the experimental design are presented in Table 2. The following second-order polynomial equation was postulated for each experimental response *Y* [21].

$$Y\;=\beta_{0}+\overset{k}{\underset{i=1}{\sum }}\beta_{i}X_{i\;}+\overset{k-1}{\underset{\begin{array}{c} i=1\\ i\;<j \end{array}}{\sum }}\overset{k}{\underset{j=2}{\sum }}\beta_{ij}X_{i}X_{j\;}\;+\overset{k}{\underset{i=1}{\sum }}\beta_{ii}X_{i}^{2}$$

where *Xi* and *Xj* represent the independent variables (*X_1_* = extraction time; *X_2_* = microwave power; and *X_3_* = temperature). *β_0_*, *β_1_*, *β_2_*, and *β_3_* denote the regression coefficients for intercept, linear, quadratic, and interaction terms, respectively; *k* denotes the number of variables. After fitting the result to the equation, we could express *Y* for the alkaloids from lotus plumule as follows:
*Y*_Higenamine_ = 782.51 + 91.38*X*_1_ + 14.86*X*_2_ + 295.64*X*_3_ – 152.04*X*_1_^2^ − 142.16*X*_2_^2^ − 135.41*X*_3_^2^*Y*_Liensinine_ = 650.95 + 117.63*X*_1_ + 25.34*X*_2_ + 315.79*X*_3_ − 174.69*X*_1_^2^ − 139.55 *X*_2_^2^*Y*_Dauricine_ = 1893.87 + 897.97*X*_3_*Y*_Isoliensinine_ = 60.41 + 18.91*X*_1_ + 39.73*X*_3_*Y*_Neferine_ = 2214.44 + 273.85*X*_1_ + 75.08*X*_2_ + 749.94*X*_3_ − 477.12*X*_1_^2^ – 405.71*X*_2_^2^ – 408.73*X*_3_^2^

Table S2 presents the ANOVA results regarding model significance and provides a summary of the statistical analysis results. The results revealed that the *p*-values derived for the developed models for the five alkaloids were < 0.001, indicating that the developed models were significant (*p* < 0.05) and that the variations in the responses could be explained by the regression equation. Lack-of-fit tests revealed nonsignificant (*p* > 0.05) values for all five alkaloids, indicating that the models could be used to predict responses. The effect of process factor on alkaloid yield is presented in the Supporting Information.

### 3.2. Verification of Optimized MAE Condition

The results of the MAE optimization process conducted using RSM along with the desirability function approach revealed that the optimal conditions for the effective MAE of the major alkaloids were as follows: Extraction time, 26 min; microwave power, 1046 W; and temperature, 120 °C. The corresponding desirability value was 0.908. On the basis of the optimization results, verification experiments were performed. Three parallel experiments were executed, after which the residuals (%) between the predicted and observed responses were calculated as follows: (Observed value−expected value)/expected value × 100%. We observed a strong correlation between the observed and predicted results (1.2–7.1% residual), confirming that the response model was adequate to reflect the expected optimization. This information is summarized in Table 3.

### 3.3. Comparison of MAE with Other Extraction Methods

As mentioned in a previous study [22], the SE approach is generally considered the benchmark for evaluation of many other extraction methods; accordingly, we used this approach in this study. Furthermore, we compared the optimized MAE conditions with the conditions of the other extraction methods, including HRE and UAE. The advantage of SE is that the sample is in repeated contact with fresh portions of the extract solvent, which promotes transfer equilibrium displacement. However, the main limitations of SE are that it involves a time-consuming solid sample preparation process and wastage of a large amount of extract solvent [23]. In the HRE technique, the solvent is transferred to the sample through distillation, and extraction occurs primarily through permeation and solubilization. Similar to SE, HRE is a time-consuming process that entails increasing the mass transfer rate through heating and mixing [24]. UAE is an inexpensive, simple, and efficient alternative to SE and HRE. Ultrasound enhances the extraction effectiveness primarily because of the effects of acoustic cavitations produced in the solvent by the passage of an ultrasonic wave [25]. Although UAE predominantly entails the use of ultrasonic baths, a lack of uniformity in the distribution of ultrasound energy and decrease in power with time may decrease the experimental repeatability and reproducibility of this extraction approach [26].

In addition to the aforementioned extraction techniques, MAE, a new extraction technique as well as a powerful sample pretreatment technique, is a potential and powerful alternative to conventional techniques for extracting alkaloid compounds from medicinal plant samples [27,28]. According to our results, MAE not only exhibited superior extraction efficiency to the other extraction methods but also required a shorter time and less solvent consumption than did SE and HRE. These results demonstrate that the optimized MAE technique constitutes a promising extraction method that offers improved efficiency by reducing the extraction time [29]. Furthermore, we observed that MAE engendered a significantly higher higenamine yield (946.3 ± 86.5 μg/g) than did SE and UAE (659.5 ± 40.9 and 671.0 ± 74.6 μg/g, respectively), but the difference in yield between MAE and HRE was nonsignificant. For liensinine yields, MAE provided a higher yield (988.6 ± 35.2 μg/g) than did the other methods. MAE also provided the highest dauricine yield (2990 ± 148.8 μg/g), but we observed no significant difference between its yield and those provided by the HRE and UAE processes, except for the SE process. Although MAE provided the highest isoliensinine yield (115.8 ± 16.1 μg/g), we observed no significant difference in yield among all extraction methods. In addition, MAE engendered a significantly higher neferine yield (2601.5 ± 80.0 μg/g) than did SE and UAE, but the observed yield did not differ significantly between MAE and HRE (Table 4). On the other hand, nuciferine has not been detected in lotus plumule using all of the extraction methods.

### 3.4. Quantitative Determination of Lotus Plumule Products

#### 3.4.1. HPLC Validation

The coefficients of determination (*R^2^*) of the calibration curves for all analytes were higher than 0.999 (Table 5). The extremely low LOD and LOQ values observed for the six compounds indicate that the analytical method had excellent sensitivity. Regarding selectivity, peaks with acceptable S/N ratios were obtained at the retention times of all investigated alkaloids, with no interfering peaks being observed at the respective retention times in the blank sample. The intra-day RSD values for the six compounds were less than 4.1%, and the RE values for accuracy varied between −3.0% and 6.9%. The inter-day RSD values for the six compounds were less than 2.4%, and the RE values for accuracy varied between −3.2% and 7.0% (Table S3). All accuracy and precision values, including LOQ, were within acceptable limits.

#### 3.4.2. Quantitative Determination of Lotus Plumule Products

**Figure 1:**
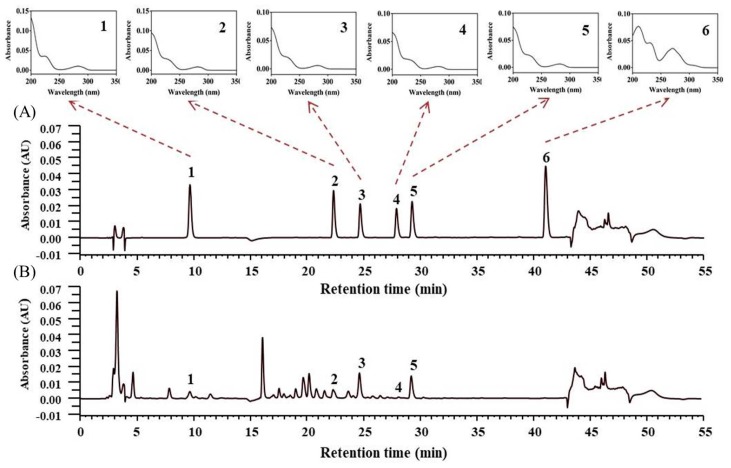
The high-performance liquid chromatography with a diode-array detector (HPLC-DAD) chromatograms of (**A**) the mixed standard solution and (**B**) lotus plumule concentrated powder using microwave-assisted extraction with 20 time dilution. 1: Higenamine; 2: Liensinine; 3: Dauricine; 4: Isoliensinine; 5: Neferine; 6: Nuciferine.

We developed a method for simultaneously identifying six lotus plumule components—namely higenamine, liensinine, dauricine, isoliensinine, neferine, and nuciferine—through gradient HPLC. Each of these authentic herbal components at a concentration of 10.0 μg/mL could be adequately separated. All alkaloids were identified through a comparison of their retention times and UV spectra with those of a standard solution. Figure 1 depicts the HPLC-DAD chromatograms of the mixed standard solution and the concentration of alkaloids in lotus plumule concentrated powder (HEP-3). Table 6 presents the concentrations of the major alkaloids in the lotus plumule products. Nuciferine was not detected in any of the samples. Among all HEPs examined in the present study, HEP-3 had the highest concentration of higenamine (approximately 872.2 μg/g). However, the concentration of the five components identified in the available lotus plumule products varied from provider to provider.

#### 3.4.3. Quality Evaluation of Lotus Plumule Products by HCA and PCA

**Figure 2:**
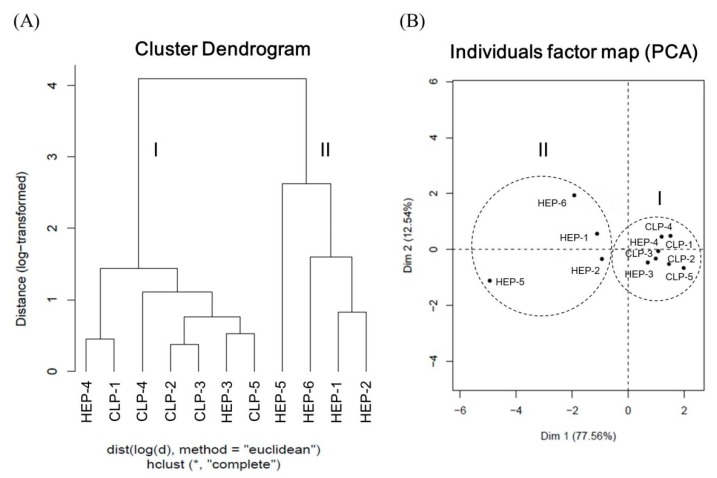
(**A**) Dendrogram of hierarchical cluster analysis and (**B**) score plot of principal component analysis for the 11 samples of lotus plumule products in Taiwan. HEP: Herbal extract product; CLP: Crude lotus plumule.

HCA is a statistical method for finding relatively homogeneous clusters of cases based on measured characteristics. We performed HCA on 11 batches of samples on the basis of the concentrations of the five alkaloids. The HCA results are presented in Figure 2A. According to the results, the samples were categorized into two broad groups: Cluster I, comprising HEP-4, CLP-1, CLP-4, CLP-2, CLP-3, HPE-3, and CLP-5 with a total alkaloid concentration of more than 6.5 mg/g; and cluster II, comprising HEP-5, HEP-6, HEP-1, and HEP-2 with a relatively low total alkaloid concentration. Particularly, HEP-5 contained the lowest content of total alkaloid and exhibited the largest distance from the others in cluster II. As revealed by the results, all CLPs belonged to cluster I because they are plant drugs occurring under fresh conditions. By contrast, HEPs are concentrated powders prepared through a series of processes, namely identification, traditional decoction, concentrating, and blending with corn starch for spray-drying. These diversified preparation steps could result in the differentiation of the alkaloids in the products. In this study, HEP-3 contained the highest concentration of total alkaloids and exhibited the largest distance from the other HEPs. PCA was also used to assess the ability to discriminate all components. Accordingly, the 11 samples were further analyzed and classified through PCA. The scatter plot of the results is presented in Figure 2B, where the distribution distances between the samples represent the similarities and differences between the samples. We observed that two dimensions accounted for 90.1% of the total variance: Dimension 1 (77.56%) and dimension 2 (12.54%). The samples were thus clustered into two groups, with cluster I having a relatively close distribution. These results were generally consistent in agreement with the HCA results.

### 3.5. Human Study

We conducted a human study to analyze the total higenamine (free form and glucuronide-conjugated form) concentration in urine specimens obtained after the oral administration of lotus plumule extract powder (HEP-3) containing 679.6 μg of higenamine per dose. The product label recommended a daily intake of 1.0–2.5 g of concentrated powder. Accordingly, we divided the recommended dose into three single doses of 0.8 g each. The participants (*n* = 6) consumed the product three times a day for three consecutive days. To evaluate the immediate concentration of higenamine in urine after the consumption of a single dose, urine samples were collected 1, 2, and 3 h after the first dose administration. In addition, to evaluate the cumulative higenamine concentration in the urine samples, urine specimens were collected 1, 2, and 3 h after the second, third, sixth, and ninth doses. The higenamine concentrations were determined by HPLC-MS/MS. 

**Figure 3:**
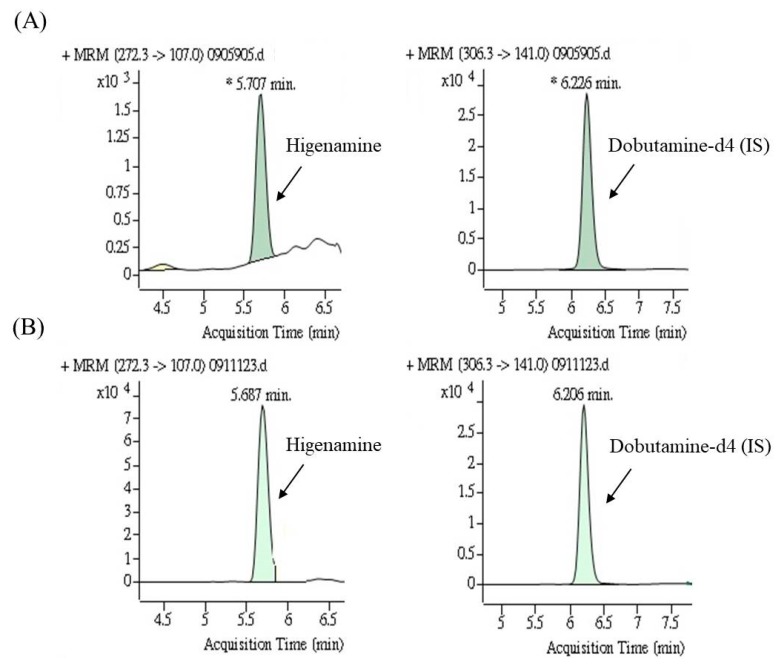
The multiple reaction monitoring (MRM) chromatograms of higenamine and internal standard: (**A**) Blank urine sample spiked with 0.5 ng/mL of higenamine and (**B**) the urine sample after consuming herbal extract product (HEP-3) by a participant.

**Figure 4:**
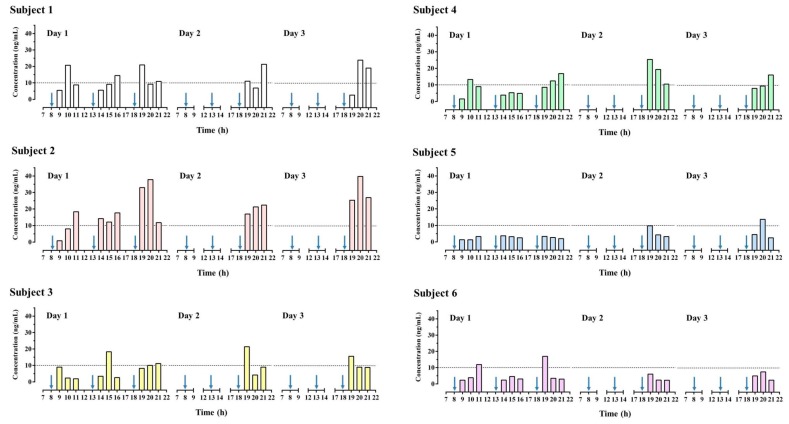
Urinary concentration-time profile of higenamine in participants received multiple administration of consuming herbal extract product (HEP-3). Arrow indicated the time of HEP-3 administration.

Figure 3 presents HPLC-MS/MS chromatograms of higenamine in a spiked urine sample and a urine sample obtained after HEP-3 consumption by a participant. The urinary concentration-time profile of higenamine is presented in Figure 4. All six participants successfully completed all aspects of the study. The results revealed that all blank urine specimens collected before the administration of lotus plumule herbal extract product do not contain higenamine, and the urinary concentrations of higenamine in four participants (subjects 1, 2, 4, and 6) reached the positivity criterion of 10.0 ng/mL within 3 h after they received the first dose. Additionally, after the administration of the third dose, the risk of reaching the positivity criterion of 10.0 ng/mL was increased. The highest higenamine concentration was observed on the second or third day among all participants except for one (subject 6). An explanation for this finding is that urinary concentrations of higenamine may have accumulated with multiple dosing. Overall, the findings indicate that participants who consumed the lotus plumule-containing HEP were at a high risk of an AAF as assessed on the basis of the WADA reporting cut-off, indicating unintentional doping; however, the concentration of the alkaloid in the plumule HEP varied considerably between individuals.

The metabolic rates of higenamine are different among species. In a rabbit model, the time to peak concentration was approximately 10 min, with the terminal half-life being approximately 20 min after oral administration; moreover, the amount of glucuronide conjugate excreted in urine was approximately 20–40% of the applied doses [30]. In a dog model, the terminal half-life of higenamine after intravenous injection was 8.6 min [31]. In a human study, the mean half-life of higenamine observed after an intravenous infusion of 22.5 μg/kg was 8 min; higenamine was eliminated through both renal excretion and the liver [32]. Although several studies have described the pharmacokinetics of higenamine, information regarding the metabolic characteristics of higenamine is limited. Excretion of glucuronide conjugates might be the predominant elimination pathway of higenamine. Liang et al. investigated the glucuronidation of higenamine and proposed that UGT1A9 is the major isoenzyme responsible for the glucuronidation of higenamine in human liver microsomes [33]. Several studies have indicated a wide interindividual variability in UGT1A9 expression, which is mediated through glucuronidation [34,35]. This thus explains why the urinary concentrations of higenamine exhibited a large interindividual variability in all six participants in the present study.

The potential nutritional characteristics of lotus plumule have been increasingly revealed recently, indicating their potential application value in functional foods, cosmetics, and pharmaceuticals. Lotus seeds and their processed by-products are widely consumed over the world for their high concentration of physiologically active substances [36,37]. Yan et al. evaluated the AAF risk associated with higenamine after the administration of lotus plumule capsules [38]; they revealed that oral administration of the capsule presented a high risk of an AAF for higenamine. All participants in their study received a specific commercial capsule, and urine specimens were collected once a day. In Taiwan, nearly all lotus plumule products prescribed by Chinese medical physicians are in the form of concentrated extract powder, which is produced by Good Manufacturing Practice-certified companies. Therefore, we first surveyed the concentrations of higenamine in commercially available products of lotus plumule in Taiwan and selected those with the highest higenamine concentration for the following elimination study. Urine specimens were collected within 3 h after every dose reception on the first day, which reflected the immediate absorption and metabolic conditions of higenamine after oral administration of the concentrated extract powder of lotus plumule. In this study, we conducted a human experiment to examine urinary metabolites after multiple administrations of lotus plumule extracts. Our data indicate that consumption of lotus plumule concentrated extract powder caused higenamine levels that violated the WADA cut-off point of 10.0 ng/mL. Accordingly, the results suggest that concentrated extract products of lotus plumule be avoided during in- and out-of-competition periods. We also evaluated the AAF risk associated with higenamine after multiple administrations of the lotus plumule HEP for three consecutive days. The results indicate that athletes would be exposed to a high risk of an AAF for higenamine after short-term supplementation. Future research could investigate the metabolic profile of higenamine and study the effects of long-term microdose administration of higenamine. The oral administration of lotus plumule herbal extract product obviously poses an AAF risk of higenamine which is also found in a variety of medicinal plants. The use of these botanical products includes the oral administration and the transdermal delivery through topical application. We believe that comprehensive studies regarding higenamine contents in lotus plumule-containing products are necessary to be conducted to fully understand the potential risk of higenamine misuse for athletes. The lack of a dietary control group may be a limitation for the current study. However, as we know, athletes may drink tea, coffee, or energy drinks during their daily life, so we think that the current study design of no dietary restriction of caffeine may be a closer situation to the actual daily life of athletes.

## 4. Conclusions

This study presents a validated HPLC-DAD method for identifying and quantifying higenamine and other major bioactive alkaloids, namely liensinine, dauricine, isoliensinine, neferine, and nuciferine, in lotus plumule. Furthermore, an optimized MAE method was used to extract alkaloids from lotus plumule using RSM to screen lotus plumule products in Taiwan. HCA and PCA provided a useful basis for overall evaluation of the quality differences among different lotus plumule products. Results obtained from comparing multiple extraction methods indicate that MAE achieved the highest extraction yields for all alkaloids in lotus plumule. Moreover, high concentrations of higenamine could be found in lotus plumule. Results obtained from a human study demonstrate that urinary concentrations of higenamine in all participants with a common consumption pattern exceeded the cut-off of 10.0 ng/mL. We suggest that athletes should avoid consuming lotus plumule during in- and out-of-competition periods and pay special attention to the use of herbal products. When athletes need to use lotus plumule containing products, they should consult health professionals, such as a physician or pharmacist, to lower the misuse risk of higenamine.

## Figures and Tables

**Table 1:** The coded values and corresponding actual values of the optimization parameters used in the response surface analysis.

Code	Extraction Time (min)	Microwave Power (W)	Temperature (°C)
−1	5	500	60
0	17.5	1000	90
1	30	1500	120

**Table 2:** Experiment conditions and the extraction yields of the alkaloids for the Box–Behnken design.

Run	Factors			Extraction Yield (μg/g)				
	X_1_ (min)	X_2_ (W)	X_3_ (°C)	Higenamine	Liensinine	Dauricine	Isoliensinine	Neferine
1	5	500	90	405.1	255.2	1372.8	15.9	1008.9
2	17.5	1500	120	874.7	856.5	3015.7	131.3	2424.6
3	17.5	1000	90	750.5	744.6	2450.6	56.7	2202.1
4	17.5	1000	90	762.3	554.9	2372.6	92.5	1991.5
5	30	1000	120	912.6	835.4	2413.5	106.7	2315.6
6	17.5	1000	90	935.4	761.9	3050.6	42.5	2776.5
7	17.5	500	120	677.9	667.0	2285.3	88.4	1808.9
8	5	1000	60	228.3	139.2	767.6	21.5	644.5
9	17.5	1500	60	205.4	136.7	771.3	20.3	632.4
10	30	500	90	582.3	536.6	2170.5	71.6	1611.1
11	5	1000	120	717.4	630.2	2395.1	88.7	1895.8
12	30	1000	60	122.0	50.8	529.0	33.6	458.4
13	30	1500	90	715.3	673.6	2549.0	84.7	2030.7
14	17.5	1000	90	707.8	684.9	2185.5	59.6	2169.2
15	5	1500	90	250.5	130.8	836.9	19.4	675.8
16	17.5	500	60	261.8	136.1	858.0	21.9	734.1
17	17.5	1000	90	756.5	757.7	2171.8	71.4	1933.0

X_1_: Extraction time; X_2_: Microwave power; X_3_: Temperature.

**Table 3:** Optimum of condition predicted and experimental value of response at the condition.

**Factor**	**Optimal Conditions**		
Extraction time (min)	26		
Microwave power (W)	1046		
Temperature (°C)	120		
**Responses**	**Predicted Value (μg/g)**	**Experimental Value (μg/g, *n* = 3)**	**Residual (%)**
Higenamine	935.4	946.3 ± 86.5	1.2
Liensinine	967.8	988.6 ± 35.2	2.2
Dauricine	2791.8	2990.0 ± 148.8	7.1
Isoliensinine	112.9	115.8 ± 16.1	2.5
Neferine	2523.8	2601.5 ± 80.0	3.1

**Table 4:** Comparison of the results of microwave-assisted extraction with other extraction methods.

Extraction Method	Extraction Time (min)	Extraction Yield (μg/g)				
		Higenamine	Liensinine	Dauricine	Isoliensinine	Neferine
SE	480	659.5 ± 40.9 ^a^	487.1 ± 122.1 ^a^	1806.0 ± 327.3 ^a^	85.7 ± 37.5	1699.0 ± 165.7 ^a^
HRE	240	831.5 ± 62.1 ^b^	590.8 ± 58.0 ^a^	2214.4 ± 204.0 ^a,b^	66.5 ± 3.6	2226 ± 286.0 ^b,c^
UAE	30	671.0 ± 74.6 ^a^	592.3 ± 39.1 ^a^	2299.0 ± 147.1 ^a,b^	80.8 ± 24.1	1962.9 ± 72.1 ^a,b^
MAE	26	946.3 ± 86.5 ^b^	988.6 ± 35.2 ^b^	2990.0 ± 148.8 ^b^	115.8 ± 16.1	2601.5 ± 80.0 ^c^

Solvent: 95% (v/v) ethanol; liquid/solid ratio: 20:1 mL/g. Results were expressed as mean ± standard deviation (*n* = 3). Different letters indicate a significant difference at *p* < 0.05. SE: Soxhlet extraction; HRE: Heat reflux extraction; UAE: Ultrasound-assisted extraction; MAE: Microwave-assisted extraction.

**Table 5:** Linear range, coefficient of determination, limits of detection, and limits of quantification of higenamine, liensinine, dauricine, isoliensinine, neferine, and nuciferine.

Analytes	Linear Range (μg/mL)	*R* ^2^	LOQ (μg/mL)	LOD (μg/mL)
Higenamine	0.05–25.0	0.9998	0.05	0.017
Liensinine	0.25–25.0	0.9999	0.25	0.083
Dauricine	0.25–25.0	0.9999	0.25	0.083
Isoliensinine	0.1–25.0	0.9999	0.1	0.033
Neferine	0.25–25.0	0.9999	0.25	0.083
Nuciferine	0.1–25.0	0.9997	0.1	0.033

LOQ: Limit of quantitation; LOD: Limit of detection.

**Table 6:** Alkaloid contents of lotus plumule products.

Product	Component (μg/g)					
	Higenamine	Liensinine	Dauricine	Isoliensinine	Neferine	Total
HEP-1	434.0 ± 30.4	517.3 ± 45.5	2005.3 ± 95.4	31.1 ± 3.1	1928.1 ± 80.6	4916.7 ± 84.2
HEP-2	488.0 ± 26.0	453.3 ± 52.6	1275.8 ± 93.4	37.0 ± 1.7	3679.2 ± 76.1	5933.2 ± 203.9
HEP-3	872.2 ± 19.6	649.0 ± 24.4	2315.3 ± 178.1	99.0 ± 6.3	5249.1 ± 115.7	9184.6 ± 82.8
HEP-4	809.1 ± 48.5	820.5 ± 96.5	3012.9 ± 82.1	88.9 ± 5.7	2409.9 ± 119.3	7141.4 ± 183.2
HEP-5	263.9 ± 23.7	114.7 ± 19.6	422.3 ± 28.3	14.5 ± 2.9	1029.5 ± 45.0	1844.0 ± 27.3
HEP-6	343.1 ± 12.7	1210.6 ± 59.6	1211.2 ± 58.9	20.5 ± 1.8	1276.2 ± 24.4	4061.6 ± 145.0
CLP-1	844.1 ± 49.9	726.0 ± 27.2	2102.7 ± 48.4	103.9 ± 4.5	2896.5 ± 159.2	6673.1 ± 180.1
CLP-2	643.2 ± 24.8	583.2 ± 40.1	2115.7 ± 134.2	59.1 ± 17.1	6567.3 ± 409.6	9968.6 ± 541.3
CLP-3	669.2 ± 53.3	740.0 ± 25.6	1914.2 ± 113.9	77.3 ± 1.7	6496.3 ± 330.1	9896.9 ± 463.8
CLP-4	721.9 ± 29.9	1635.0 ± 25.4	1686.6 ± 54.9	79.2 ± 5.1	7345.2 ± 300.6	11466 ± 355.2
CLP-5	969.5 ± 43.3	761.7 ± 36.9	2355.9 ± 190.3	98.0 ± 2.6	8554.3 ± 202.2	12739.4 ± 433.2

Results were expressed as mean ± standard deviation (*n* = 3). HEP: Herbal extract products; CLP: Crude lotus plumule.

## Supplementary Materials

The following are available online at https://www.mdpi.com/2072-6643/12/2/285/s1. Table S1: Validation of liquid chromatography with tandem mass spectrometry for higenamine; Table S2: Analysis of variance (ANOVA) statistics of the quadratic model for the extraction yields of the alkaloids; Table S3: Intra- and inter-day precision and accuracy of the HPLC method for the determination of higenamine, liensinine, dauricine, isoliensinine, neferine, and nuciferine. Figure S1: Response surface plot and contour plot of the interaction effects of extraction time, microwave power, and temperature on the yield of higenamine; Figure S2: Response surface plot and contour plot of the interaction effects of extraction time, microwave power, and temperature on the yield of liensinine; Figure S3: Response surface plot and contour plot of the interaction effects of extraction time, microwave power, and temperature on the yield of dauricine; Figure S4: Response surface plot and contour plot of the interaction effects of extraction time, microwave power, and temperature on the yield of isoliensinine; Figure S5: Response surface plot and contour plot of the interaction effects of extraction time, microwave power, and temperature on the yield of nuciferine.

## Abbreviations

WADA: World Anti-Doping Agency; AAF: Adverse analytical finding; DOE: Design of experiment; MAE: Microwave-assisted extraction; RSM: Response surface methodology; HCA: Hierarchical cluster analysis; PCA: Principal component analysis; HEP: Herbal extract products; BBD: Box–behnken design; HPLC: High-performance liquid chromatography; SE: Soxhlet extraction; HRE: Heat reflux extraction; UAE: Ultrasound-assisted extraction; UV: Ultraviolet; QC: Quality control; LOQ: Limit of quantification; LOD: Limit of detection; S/N: Signal-to-noise; ANOVA: Analysis of variance; RSD: Relative standard deviation; RE: Relative error; HEP: Herbal extract product; CLP: Crude lotus plumule; IS: Internal standard; HPLC-MS/MS: High-performance liquid chromatography with tandem mass spectrometry; HPLC-DAD: High-performance liquid chromatography with a diode-array detector; MRM: Multiple reaction monitoring.
