# Supplementary material for: Potential Risk of Higenamine Misuse in Sports: Evaluation of Lotus Plumule Extract Products and a Human Study
Source: Nutrients. 2020 Jan 21;12(2):285. doi: 10.3390/nu12020285 (PMC7070534; doi:10.3390/nu12020285)
Supplement: Supplementary file 1 [file nutrients-12-00285-s001.pdf]

Table S1.

Validation of liquid chromatography with tandem mass spectrometry for higenamine

Concentration (ng/mL)	Intra-day			Inter-day			Recovery	
	Mean ± SD	RSD (%)	RE (%)	Mean ± SD	RSD (%)	RE (%)	Mean ± SD	RSD (%)
1	0.90 ± 0.01	0.6	89.9	0.90 ± 0.03	3.1	89.7	73.79 ± 0.81	1.1
2.5	2.24 ± 0.03	1.4	89.4	2.40 ± 0.15	6.3	96.2	82.69 ± 0.18	0.2
5	4.46 ± 0.04	0.9	89.3	4.68 ± 0.15	3.2	93.6	75.33 ± 0.37	0.5

Results were expressed as mean ± standard deviation (n = 3).

Table S2.
Analysis of variance (ANOVA) statistics of the quadratic model for the extraction yields of the alkaloids.

Effect	Sum of squares	d.f.	Mean square	*F*-value	*p*-value	Remarks
Higenamine						
Model	1057862.65	6	176310.44	12.89	0.0003	Significant
Residual	136745.14	10	13674.51			
Lack-of-fit	105678.07	6	17613.01	2.27	0.2239	Not significant
Pure error	31067.07	4	7766.77			
Cor total	1194607.79	16				
Liensinine						
Model	1136837.51	5	227367.50	12.28	0.0003	Significant
Residual	203614.34	11	18510.39			
Lack-of-fit	173192.53	7	24741.79	3.25	0.1356	not significant
Pure error	30421.81	4	7605.45			
Cor total	1340451.86	16				
Dauricine						
Model	6450779.40	1	6450779.40	19.83	0.0005	Significant
Residual	4880747.45	15	325383.16			
Lack-of-fit	4366735.92	11	396975.99	3.09	0.1437	not significant
Pure error	514011.52	4	128502.88			
Cor total	11331526.85	16				
Isoliensinine						
Model	15487.02	2	7743.51	24.27	< 0.0001	Significant
Residual	4467.35	14	319.10			
Lack-of-fit	3065.19	10	306.52	0.87	0.6093	not significant
Pure error	1402.17	4	350.54			
Cor total	19954.37	16				
Neferine						
Model	7738147.77	6	1289691.29	10.65	0.0007	Significant
Residual	1210630.02	10	121063.00			
Lack-of-fit	763605.21	6	127267.54	1.14	0.4711	not significant
Pure error	447024.81	4	111756.20			
Cor total	8948777.78	16				

Table S3.

Intra- and inter-day precision and accuracy of the HPLC method for the determination of higenamine, liensinine, dauricine, isoliensinine, neferine, and nuciferine.

Compound	Concentration	Intra-day (*n* = 6)			Inter-day (*n* = 6)		
	(μg/mL)	Mean ± SD	RSD(%)	RE (%)	Mean ± SD	RSD(%)	RE (%)
Higenamine	0.1	0.11 ± 0.004	4.1	6.9	0.11 ± 0.003	2.4	6.5
	1.0	1.01 ± 0.02	1.5	0.8	0.97 ± 0.01	1.4	-3.2
	10.0	10. 08 ± 0.04	0.4	0.8	10.12 ± 0.07	0.7	1.2
Liensinine	0.4	0.42 ± 0.004	0.9	5.1	0.43 ± 0.01	1.7	7.0
	2.5	2.53 ± 0.04	1.7	1.1	2.56 ± 0.02	0.6	2.5
	10.0	10.03 ± 0.08	0.8	0.3	10.05 ± 0.08	0.8	0.5
Dauricine	0.4	0.41 ± 0.01	2.9	2.3	0.42 ± 0.002	0.5	5.9
	2.5	2.48 ± 0.03	1.2	-0.1	2.51 ± 0.03	1.1	0.4
	10.0	10.01 ± 0.12	1.2	0.1	9.98 ± 0.10	1.0	-0.2
Isoliensinine	0.25	0.24 ± 0.004	1.8	-3.0	0.25 ± 0.005	1.9	-2.1
	2.5	2.51 ± 0.04	1.5	0.4	2.56 ± 0.03	1.3	2.2
	10.0	10.15 ± 0.05	0.5	1.5	10.56 ± 0.06	0.6	2.5
Neferine	0.4	0.39 ± 0.005	1.2	-1.3	0.39 ± 0.01	1.7	-3.1
	2.5	2.52 ± 0.05	1.8	0.7	2.45 ± 0.02	0.7	-2.0
	10.0	10.06 ± 0.04	0.4	0.6	10.19 ± 0.07	0.7	1.9
Nuciferine	0.25	0.25 ± 0.007	2.8	-0.1	0.25 ± 0.005	1.8	0.8
	2.5	2.48 ± 0.01	0.4	-0.6	2.44 ± 0.01	0.5	-2.3
	10.0	10.07 ± 0.06	0.6	0.7	10.12 ± 0.08	0.8	1.2

SD: standard deviation.

Response surface plot and contour plot showing effects of extraction variables on yield of lotus plumule alkaloids

To understand the interaction of the three factors as they affect the yields of the five alkaloids, the 3D response surface and contour plots for the analytes were shown in **Figure S1-S5**. The results show the response surface and contour plots for the major alkaloids. As we can see, the results are remarkably consistent among the yield of the alkaloids except for isoliensinine. The yield of higenamine, liensinine, dauricine, and neferine gradually increased with an increase of extraction time and microwave power and then declined. We also find that with an increase in temperature, the extraction yields gradually increased in the results of the interaction between extraction time with temperature and microwave power with temperature. Actually, the yield of these four alkaloids was all markedly affected by the temperature ($p<0.0005$). These results indicated that the optimized conditions for extracting higenamine are an extraction time of 20.8 min, a microwave power of 1083 W, and a temperature of 112.7 °C. For liensinine, the optimized conditions for extracting liensinine are an extraction time of 15.2 min, a microwave power of 703.8 W, and a temperature of 119.8 °C. For dauricine, the optimized conditions for extracting dauricine are an extraction time of 21.9 min, a microwave power of 1302.0 W, and a temperature of 120.0 °C. For neferine, the optimized conditions for extracting neferine are an extraction time of 21.1 min, a microwave power of 1046.6 W, and a temperature of 117.4 °C. Regarding the isoliensinine extraction yield, the yield reached the maxima when extraction time and temperature approached on the top of the range. With an increase of microwave power, the yield of isoliensinine increased and then declined. These results showed that the optimized conditions for extracting isoliensinine are an extraction time of 30.0 min, a microwave power of 1000 W, and a temperature of 120°C.

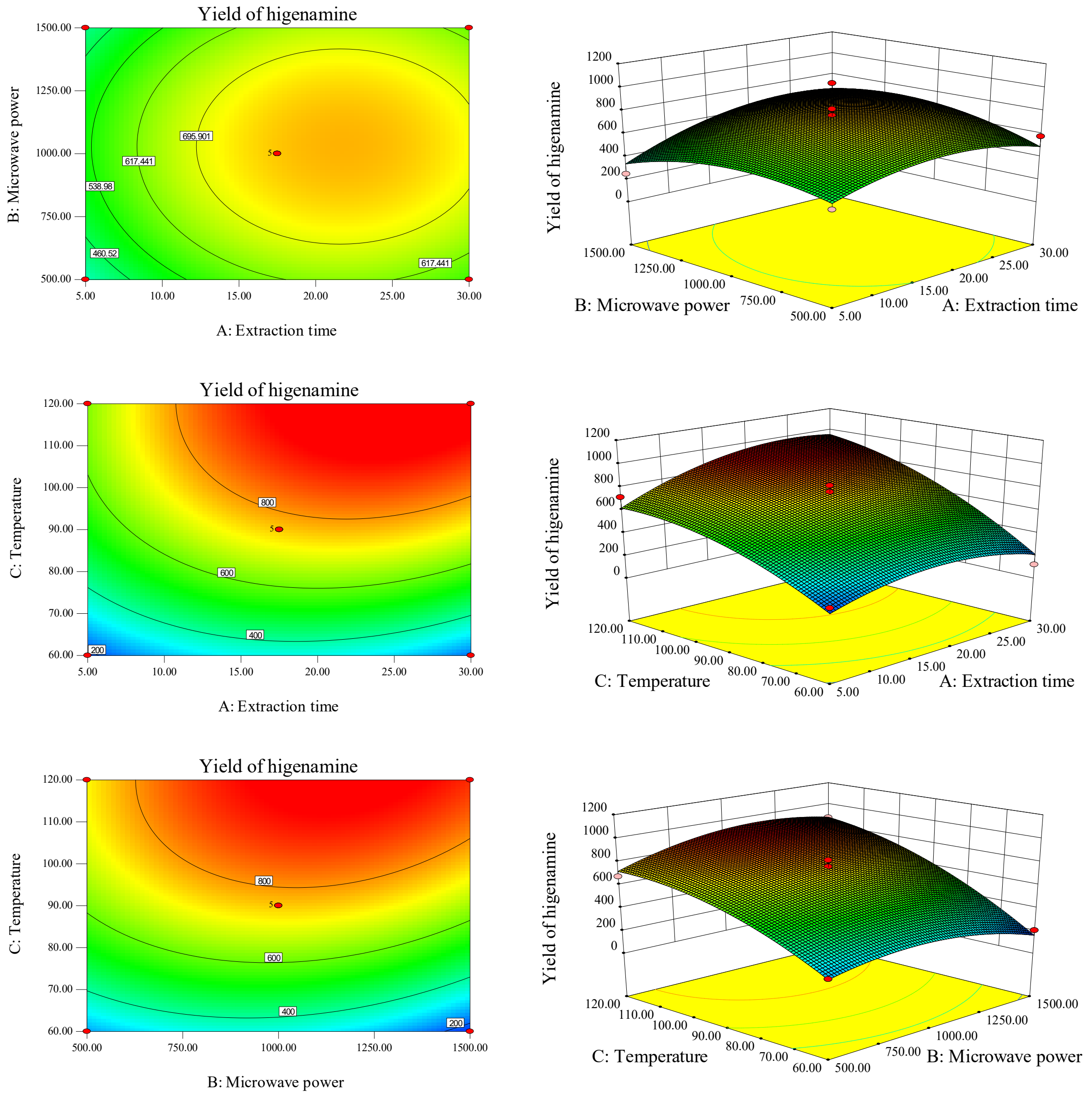

Figure S1. Response surface plot and contour plot of the interaction effects of extraction time, microwave power, and temperature on the yield of higenamine.

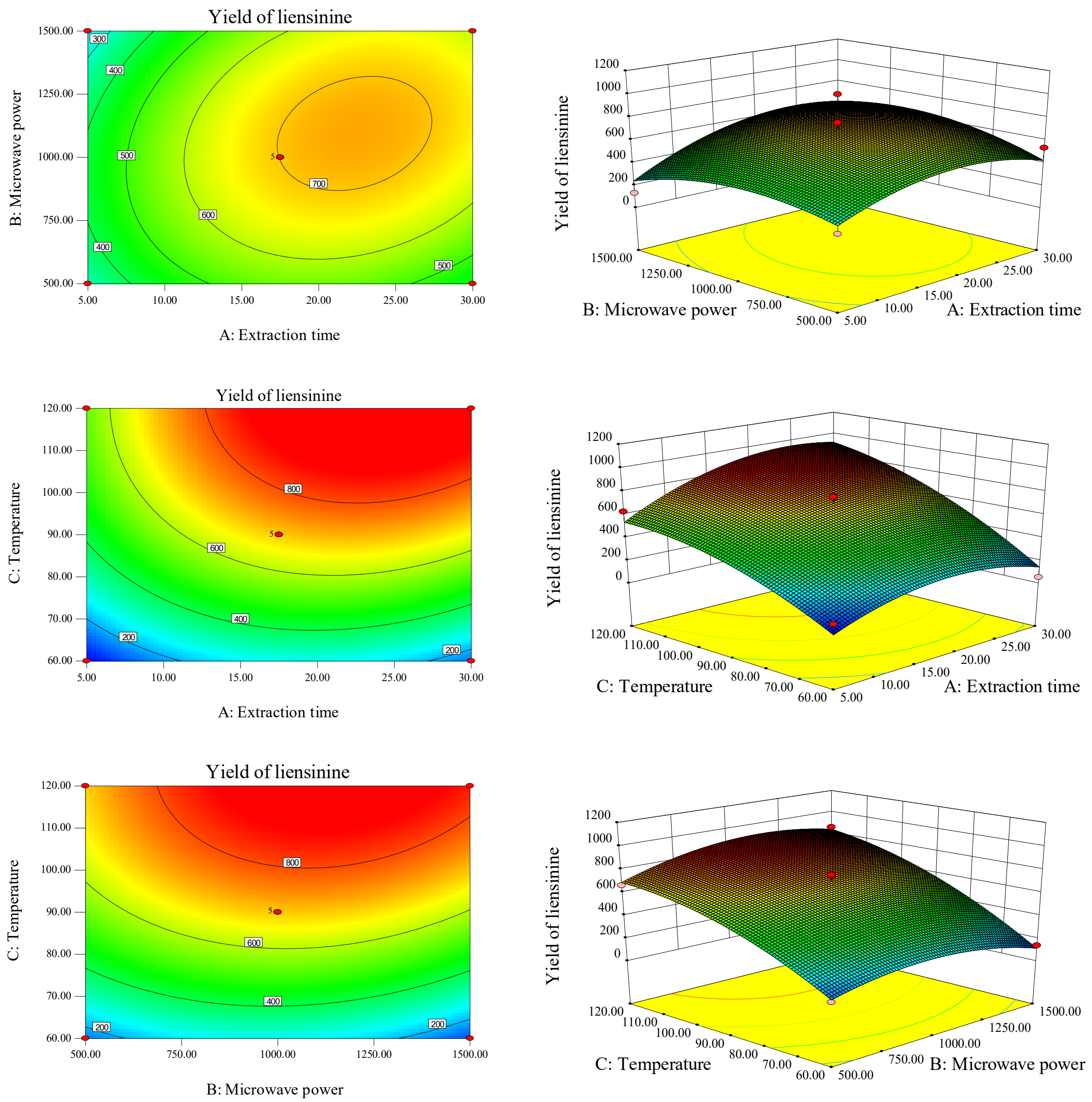

Figure S2. Response surface plot and contour plot of the interaction effects of extraction time, microwave power, and temperature on the yield of liensinine.

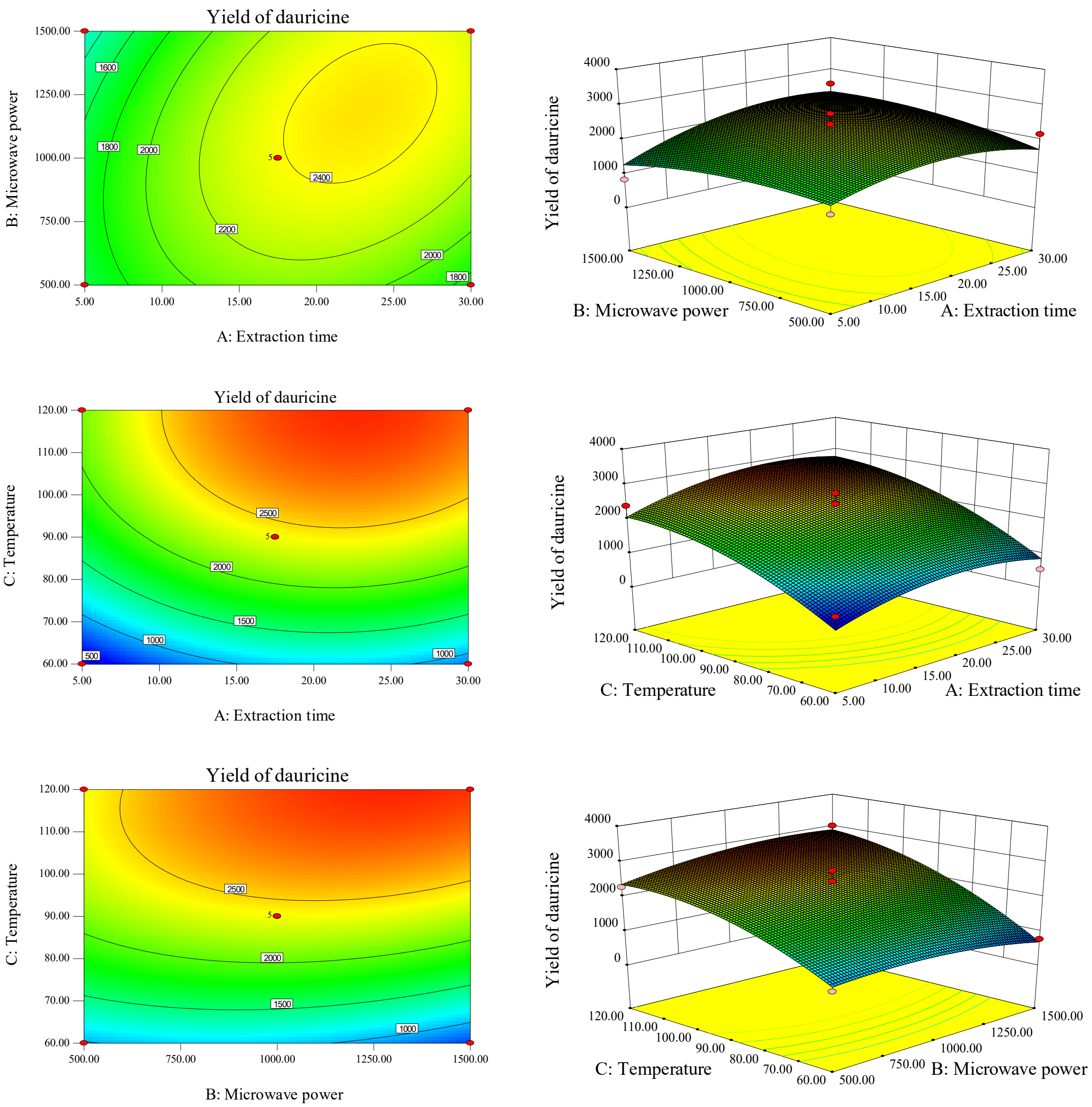

Figure S3. Response surface plot and contour plot of the interaction effects of extraction time, microwave power, and temperature on the yield of dauricine.

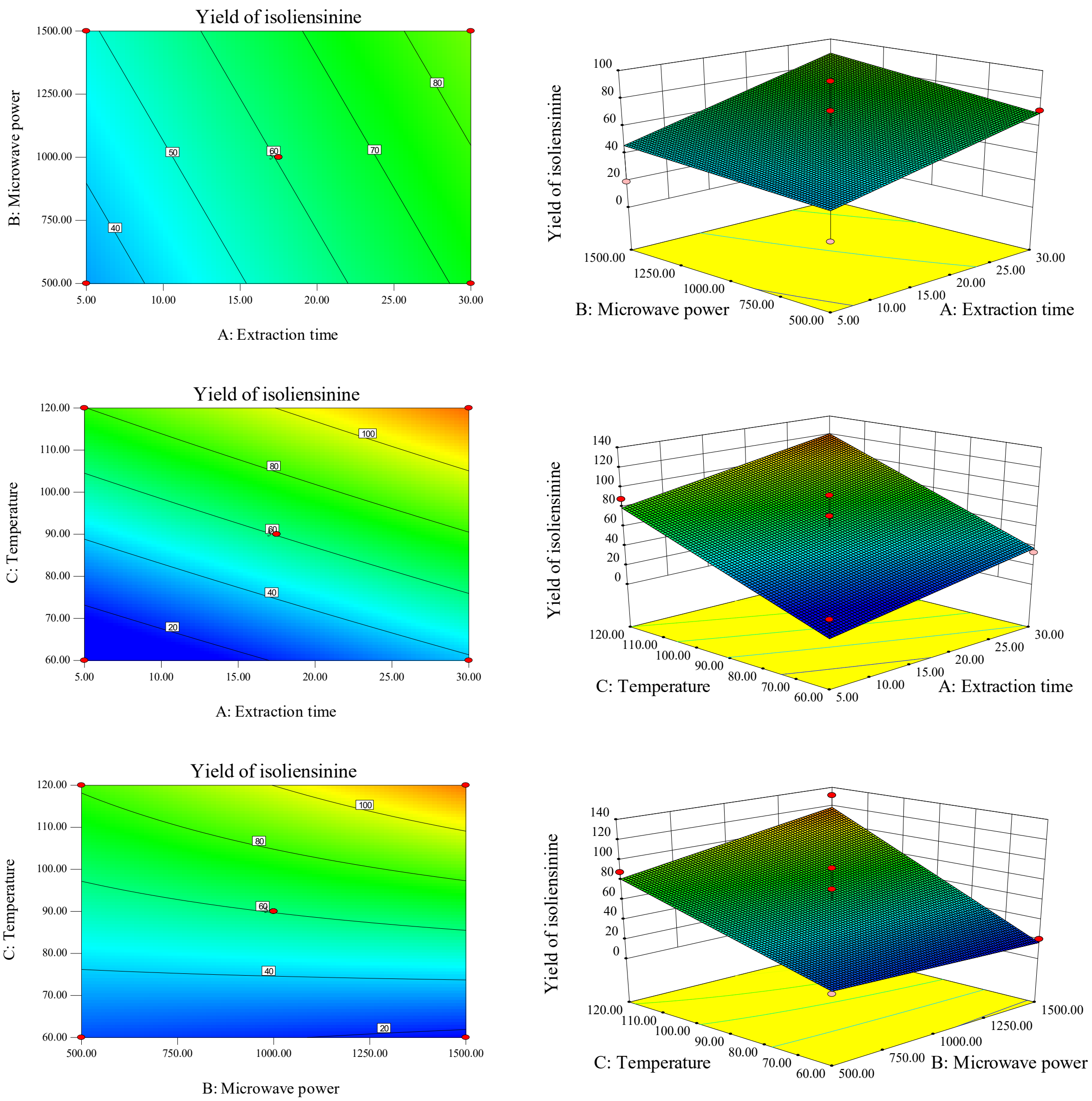

Figure S4. Response surface plot and contour plot of the interaction effects of extraction time, microwave power, and temperature on the yield of isoliensinine.

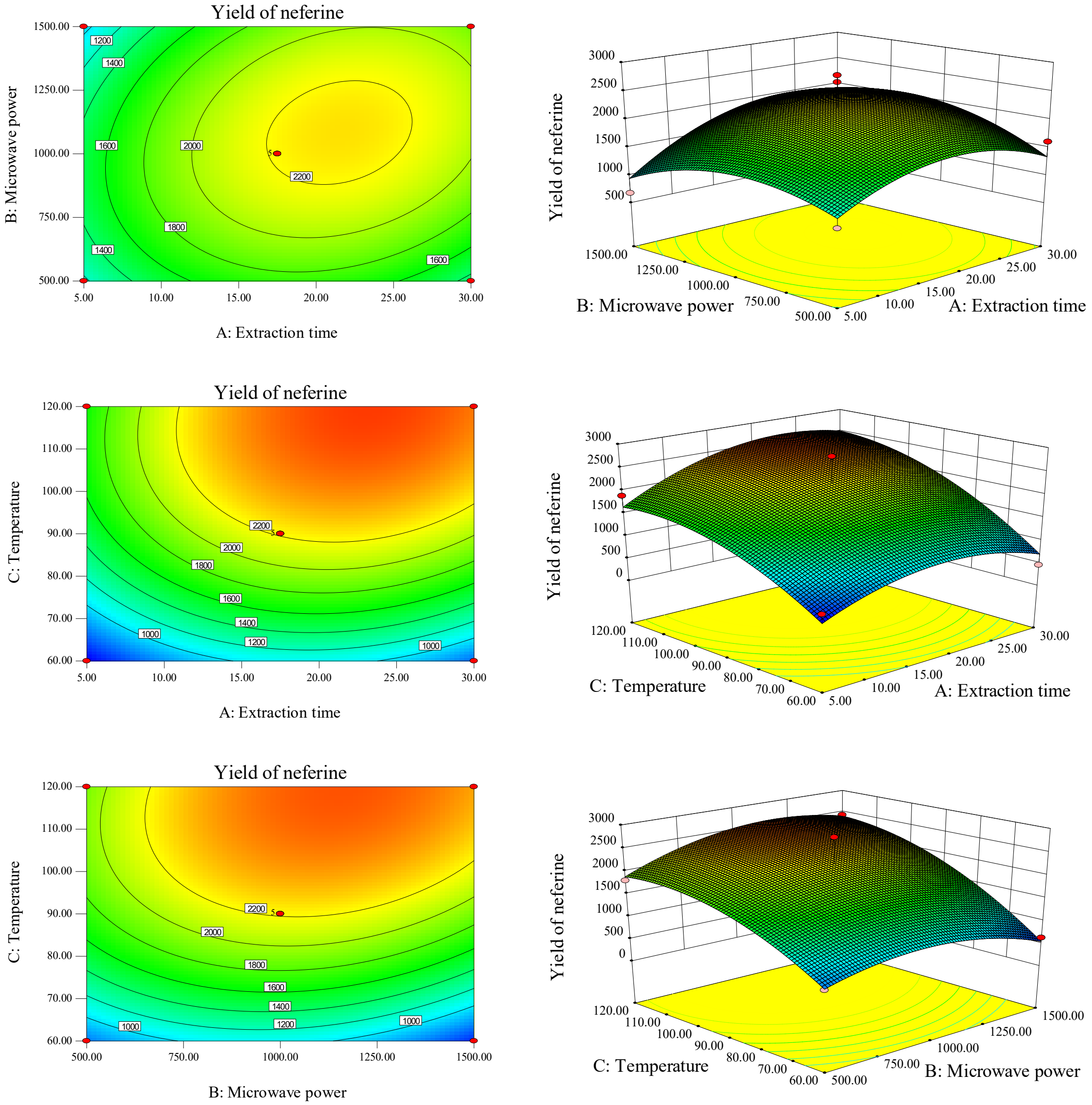

Figure S5. Response surface plot and contour plot of the interaction effects of extraction time, microwave power, and temperature on the yield of nuciferine.
